# Saving Lives Together: A Qualitative Evaluation of the Saving Mothers, Giving Life Public-Private Partnership

**DOI:** 10.9745/GHSP-D-18-00264

**Published:** 2019-03-11

**Authors:** Anne Palaia, Lauren Spigel, Marc Cunningham, Ann Yang, Taylor Hooks, Susan Ross

**Affiliations:** a Bureau for Global Health, U.S. Agency for International Development, Washington, DC, USA.; b ICF, Fairfax, VA, USA. Now with Ariadne Labs, Boston, MA, USA.

## Abstract

**Background:**

Public-private partnerships (PPPs) have garnered appeal among governments around the world, making impressive contributions to health resource mobilization and improved health outcomes. Saving Mothers, Giving Life (SMGL), a PPP aimed at reducing maternal deaths, was born out of the need to mobilize new actors, capitalize on diverse strengths, and marshal additional resources. A qualitative study was initiated to examine how the SMGL partnership functioned to achieve mortality reduction goals and foster country ownership and sustainability.

**Methods:**

We purposively selected 57 individuals from U.S. and global public and private partner organizations engaged in SMGL in Uganda and Zambia for qualitative in-depth interviews. Representative selection was based on participant knowledge of partner activities and engagement with the partnership at various points in time. Of those invited, 46 agreed to participate. Transcripts were double-coded, and discordant codes were resolved by consensus.

**Results:**

Several recurring themes emerged from our study. Perceived strengths of the partnership included goal alignment; diversity in partner expertise; high-quality monitoring, evaluation, and learning; and strong leadership and country ownership. These strengths helped SMGL achieve its goals in reducing maternal and newborn mortality. However, uncertainty in roles and responsibilities, perceived power inequities between partners, bureaucratic processes, a compressed timeline, and limited representation from ministries of health in the SMGL governance structure were reported impediments.

**Conclusion:**

While SMGL faced many of the same challenges experienced by other PPPs, local counterparts and the SMGL partners were able to address many of these issues and the partnership was ultimately praised for being a successful model of interagency coordination. Efforts to facilitate country ownership and short-term financial sustainability have been put in place for many elements of the SMGL approach; however, long-term financing is still a challenge for SMGL as well as other global health PPPs. Addressing key impediments outlined in this study may improve long-term sustainability of similar PPPs.

## Abstract (precis)

Overall, the Saving Mothers, Giving Life partnership was praised as a successful model for interagency coordination. Key strengths included diversity in partner expertise, high-quality monitoring and evaluation, strong leadership, and country ownership. Uncertainty about partner roles and responsibilities, perceived power inequities between partners, bureaucratic processes, and limited Ministry of Health representation in the governance structure were some challenges that, if addressed by similar public-private partnerships under development, may improve long-term partnership success.

## BACKGROUND

Public-private partnerships (PPPs), generally defined as “cooperative institutional arrangements between public and private sectors,” have garnered appeal among governments around the world.^1^ In the field of international health, global health PPPs, a subset of PPPs, have made impressive contributions to national health policies and agendas, health advocacy, health resource mobilization, and improved health outcomes.^2^^,^^3^ Global health PPPs, defined as “relatively institutionalized initiatives, established to address global health problems, in which public and for-profit private sector organizations have a voice in collective decision-making,”^2^^–^^4^ have mushroomed since the late 1990s with an estimated 10 new partnerships being formed annually.^4^ The proliferation of global health PPPs has triggered the need for research to better understand the barriers and facilitators to goal achievement within partnerships.^5^

The Saving Mothers, Giving Life (SMGL) partnership was born out of the U.S. Global Health Initiative (GHI), an overarching approach to U.S. global health policy introduced in 2009 that provided a guiding framework to strengthen and streamline existing U.S. global health programs. Recognizing the complexities, interconnectedness, and urgency of women's sexual and reproductive health issues, the GHI emphasized local ownership, integration of health sectors, and gender equality to improve the efficiency and effectiveness of global health programs. The goal of SMGL was to establish a highly-visible maternal health program that capitalized on diverse yet complementary strengths and marshalled additional resources. Along with financial support, each SMGL founding partner brought unique skills and expertise to the initiative:

- The U.S. Centers for Disease Control and Prevention (CDC) and the United States Agency for International Development (USAID) led the initiative for the United States Government (USG), with support from the Department of State and Department of Defense, to provide existing on-the-ground support for country maternal/newborn health and HIV/AIDS programs and technical expertise in health and development
- The Government of Norway made a commitment to expand the global focus on maternal mortality reduction and provided thought leadership in information systems
- Merck for Mothers guided the strategic direction of the initiative, supported on-the-ground program implementation and evaluation, worked with partners to raise public awareness, and served as the Secretariat
- The American College of Obstetricians and Gynecologists (ACOG) provided thought leadership in implementation science, clinical intervention, and technical skill building
- Every Mother Counts (EMC) provided leadership in communication strategies and emergency transportation and referral systems
- Project C.U.R.E. procured donated hospital supplies and equipment for SMGL-supported districts

Additional information on partner roles and responsibilities is available in Table 1.

**TABLE 1.** Saving Mothers, Giving Life Partner Roles and Responsibilities, by Geographic Scope

Geographic Scope			
Partner	Global	Uganda	Zambia
American College of Obstetrics and Gynecology	Thought leadership on implementation science	Mentorship training of OB/GYN society (USAID)	Support national adoption of uterine balloon tamponade (USAID-supported)
Every Mother Counts	Advocacy/media campaigns Co-Chair of Communication Committee	Fund emergency transportation and referral systems	
Government of Norway	Thought leadership on health information systems	Funded Project C.U.R.E. to provide supplies/equipment	
Merck for Mothers	Support Phase 1 Secretariat Support website/communication	Strengthen local private health care providers in Uganda	Develop entrepreneurial approaches for maternity waiting homes Support Zambia endline census
Project C.U.R.E.	Co-Chair of New Partnership Committee	Ensure availability of critical supplies/equipment for services (funded by USAID and Government of Norway)	
USAID (lead USG agency)	Lead SMGL for USG Support SMGL Secretariat for Phase 2 Co-Chair and fund M&E Working Group Lead MNH technical oversight, support country programs	USAID Mission support for postpartum family planning, voucher programs, private-sector services, and quality assurance	USAID Mission support for behavior change efforts, technical training and mentoring, and district coordinators
State/OGAC	Technical guidance and funding to country teams, outside of the Country Operational Plan funds	CDC and USAID Missions provide HIV/AIDS technical oversight and support to country programs	
CDC	Lead M&E efforts for the SMGL initiative, including cross-country analysis Co-Chair M&E Working Group (funded by USAID)	Lead M&E activities for the country including RAMOS, HFAs, POMS, MDSR, and BABIES (funded by OGAC)	Lead M&E activities for the country census, HFA, MDSR (funded by OGAC)
U.S. Department of Defense	N/A	N/A	Support work with 7 government military health facilities, including upgrading maternity wards and operating rooms Construct 7 maternity waiting homes
Peace Corps	Develop training curriculum on MCH for Peace Corps volunteers	N/A	Support community health workers located in SMGL districts

Abbreviations: BABIES, Birth Weight and Age-at-Death Boxes for Intervention and Evaluation System; CDC, U.S. Centers for Disease Control and Prevention; HFA, health facility assessment; M&E, monitoring and evaluation; MCH, maternal and child health; MDSR, maternal death surveillance and response; MNH, maternal and neonatal health; OB/GYN, obstetrics and gynecology; OGAC, Office of the U.S. Global AIDS Coordinator; POMS, Pregnancy Outcomes Monitoring Survey; RAMOS, Reproductive Age Mortality Study; SMGL, Saving Mothers, Giving Life; USAID, United States Agency for International Development; USG, United States Government.

In July 2012, then Secretary of State Hillary Clinton announced SMGL as a 5-year initiative. The proof-of-concept phase was to be implemented for 1 year in Uganda and Zambia. If the SMGL model successfully decreased maternal mortality, it was anticipated that the model would be expanded. The number of countries that would ultimately be involved in SMGL varied according to source, from 3 to 10. In January 2014, USAID announced SMGL would be scaled up nationally in Uganda and Zambia and move into 3 more sub-Saharan African countries. By 2017, SMGL was working in 3 countries: Nigeria, Uganda, and Zambia.

Given the continued interest for networked approaches to solving global health problems and the importance of “partnership” as the operational basis for SMGL, a qualitative evaluation was conducted to examine: how the SMGL partnership contributed to achieving its stated objective; how it was organized and how it functioned; and how it fostered country ownership and sustainability in the long term. This article focuses on partnership efforts in Uganda and Zambia where the initiative has ended and outcomes and impacts are available. Results from Nigeria are forthcoming.

## SMGL GOVERNANCE AND GOALS

After partners were recruited and the memorandum of understanding signed, the governance structure was established. Each of the partners designated a representative to the 7-member Leadership Council, SMGL's governing body. Two seats were filled with USG representatives (from the Office of the U.S. Global AIDS Coordinator [OGAC] and USAID), and the other 5 seats were filled by the remaining partners. The Leadership Council met quarterly and was supported by 7 committees and working groups: operations, partnership, monitoring and evaluation, communications, publications, technical, and Phase 2 planning. The Leadership Council functioned as a coordinated effort to address emerging issues and steer the SMGL initiative toward its goal. Topics addressed included addition of new countries into the SMGL partnership; approval of new partners, country budgets, and evaluation and dissemination plans; and timeline and programming changes during Phase 2 to address challenges identified in Phase 1. Supported by Merck for Mothers, the Secretariat was established to execute the decisions of the Leadership Council, coordinate the inputs of USG and non-USG partners, provide oversight for country implementation and monitoring and evaluation activities, and develop yearly country budgets and work plans with stakeholders to promote timely funding (Figure 1). During the second phase, the Secretariat shifted to USAID.

**FIGURE:**
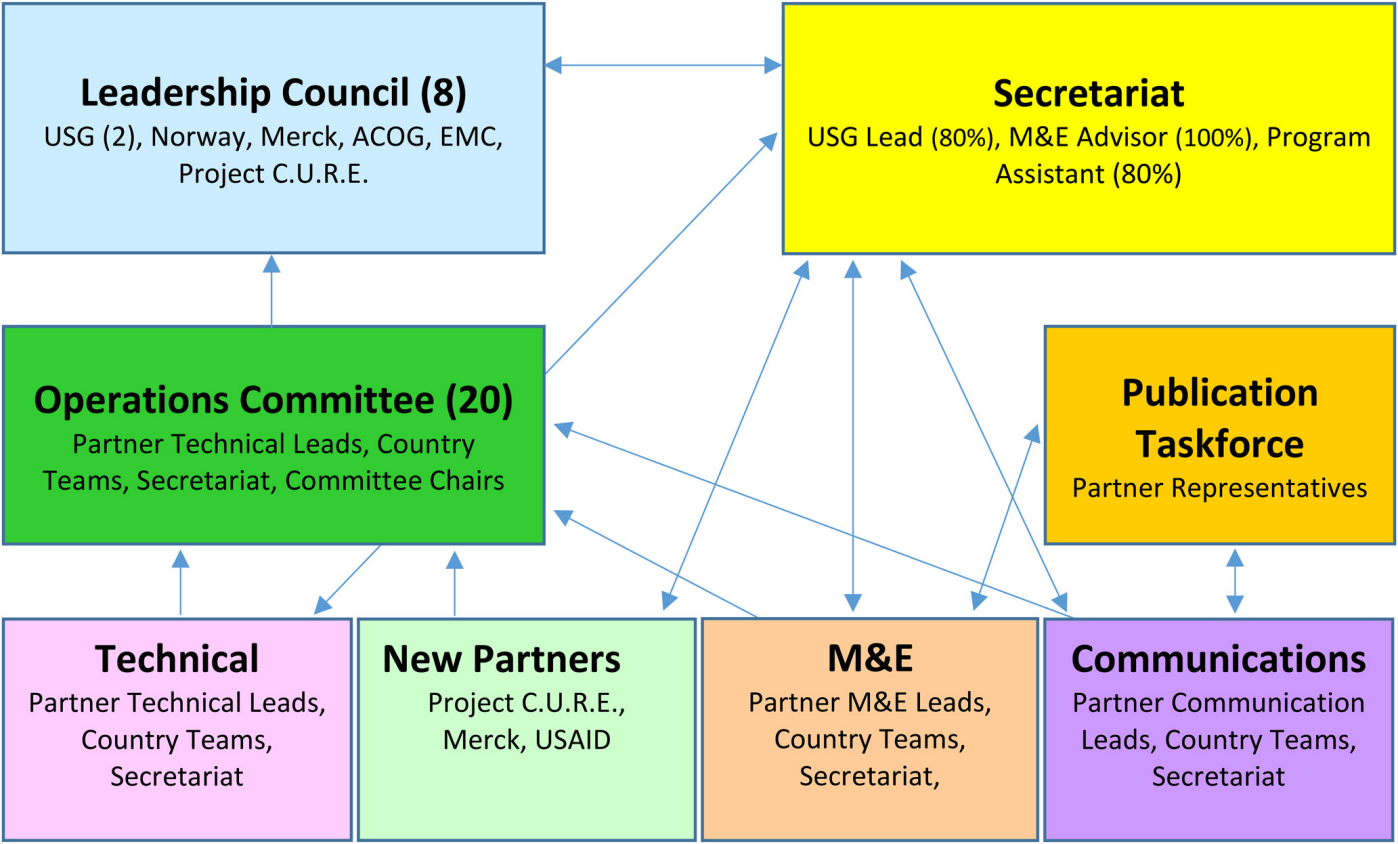
Saving Mothers, Giving Life Partnership Governance Structure Abbreviations: ACOG, American College of Obstetrics and Gynecology; EMC, Every Mother Counts, M&E, monitoring and evaluation; USAID, United States Agency for International Development; USG, United States Government.

The founding partners pledged over US$200 million in cash and in-kind contributions over 5 years.^6^ The Secretariat began requiring quarterly submission of expenditures from each partner in 2013.^7^ Over the first 33 months of operation (January 2012–September 2014), the partners contributed 23% of the total SMGL pledge and cash flow to implementing partners was erratic. In late 2014, partners were asked to reconsider and revise their initial pledges (both in-kind and cash) with the expectation that these revised pledges would be spent down during the remaining 3 years of the initiative, fostering accountability and more predictable funding. Revised partner pledges totaled US$138 million. OGAC confirmed funding from the U.S. President's Emergency Plan for AIDS Relief (PEPFAR) for SMGL implementation in Uganda and Zambia, by year, for the 4 remaining years in decreasing tranches. Having a defined schedule for the decrease in funding from PEPFAR allowed for better planning at the SMGL Secretariat and country levels. It also meant that Uganda and Zambia would likely remain the focus of SMGL through the second phase of the initiative as OGAC was a major funder. (For more information on costs, incremental costs, and incremental cost per death averted in these 2 SMGL countries, see the companion article by Johns et al.^8^ in this supplement.)

### SMGL Partnership Goal

Initially, the proposed SMGL goal was to reduce the maternal mortality ratio by 8% in SMGL-supported districts within 1 year.^9^ Almost immediately, this target was deemed too lackluster to engender a movement that could break down siloed patterns of working among USG agencies, build global commitment, signal urgency, and drive creativity. Therefore, an aspirational goal was set at a 50% reduction in maternal deaths in SMGL-supported facilities within 1 year. This percentage, though unprecedented, was supported by mathematical modeling using the effect sizes of high-impact interventions with effective coverage of the population in the SMGL learning districts. In 2013, the SMGL goal was amended to include a 30% reduction in facility-based neonatal deaths and the time frame was expanded to September 2017. This expansion to include newborn mortality was established by the Leadership Council as interest in newborn outcomes increased globally and was also supported by similar modeling exercises.

The Saving Mothers, Giving Life partnership set an aspirational goal to reduce maternal deaths by 50% in SMGL-supported facilities within 1 year.

The SMGL initiative was divided roughly into 3 phases: Design and Planning Phase (2011–2012), Phase 1 Proof of Concept (2012–2013), and Phase 2 Scale Up and Scale Out (2013–2017). An evaluation conducted after Phase 1 of the SMGL program revealed consensus among SMGL global leaders that the partnership was “greater than the sum of its parts,” as it leveraged resources and stimulated new ideas.^10^ However, as found in prior evaluations of global health PPPs,^2^^,^^3^ the lack of clear roles and an agreed-upon operational and financing plan hindered its effectiveness and complicated planning. In addition, both USG agencies and host governments agreed that the national governments were supportive of SMGL, but they did not truly “own” the program. A number of factors hindered such leadership, including reliance on USG resources channeled outside of host-country government budgets, the understaffing of the respective Ministry of Health (MOH) positions, particularly at senior levels, and the reorganization of the Zambian MOH.

### SMGL Phase 2 Modifications

During Phase 2, changes were also made to partnership procedures and processes. First, as mentioned previously, partners had to report their quarterly contributions, which the Leadership Council reviewed, and they revised their original pledges to be more realistic with the expectation that the revised pledges would be expended by the end of the partnership. The declining tranches of PEPFAR funding facilitated MOH yearly budget negotiations around domestic funding and institutionalization.

An Operations Committee comprised of partner technical leads also was constituted to assist with implementation. Operational issues were discussed and determined at this level, with only higher-level governance issues decided by the Leadership Council. Finally, there was an attempt to increase MOH leadership in SMGL by inviting MOH representatives from Uganda and Zambia to join the Leadership Council.

In 2014, Norway made the decision to transfer its monetary pledge from SMGL to the newly organized Global Financing Facility and to become inactive in the SMGL partnership. While the redirection of funds had no immediate effect on implementation, it influenced the decision of the partnership to limit its efforts to 3 countries. Coordination and direction were key components of leadership in the SMGL partnership. The Leadership Council and the Operations Committee, with input from district-level MOHs, developed an agreed-upon model to be standardized across all the implementing partners. There was also a small Secretariat that worked with the Leadership Council, Operations Committee, the Inter-Agency Working Groups, and the MOH to ensure execution. (For more information about SMGL structure, timeline, phases, modifications, and goals, see the companion article by Conlon et al. in this supplement.^11^)

## METHODS

### Study Design

We conducted a cross-sectional qualitative study between June and December 2017, with the majority of data collected in June and July.

### Selection and Sampling

We purposively selected 57 individuals from U.S. and global public and private partner organizations engaged in SMGL Zambia and SMGL Uganda to participate in qualitative in-depth interviews. Since in-country implementing partners were not part of the SMGL governance structure and did not participate in key decisions influencing the direction of the partnership, they lacked the relevant knowledge for this line of partnership-focused inquiry. Thus, they were purposefully excluded from the study.

The sample size was estimated to include representatives from each major group, including the Leadership Council, host governments, and other donors. The sample size was also estimated to account for potential refusals and an adequate number of respondents to reach thematic saturation. Representative selection was based on participant knowledge of partner activities and engagement with the partnership at various points in time. In total, 46 individuals agreed to participate (Table 2). Informed consent was obtained for each interview. When possible, written consent was obtained. When interviews were conducted via phone and scanning consent documents posed a burden on the subject, full, recorded verbal consent was obtained.

**TABLE 2.** Participant Sampling Groups

	Sampled	Participated*	Interviewed on the Governance Framework	Interviewed for Country Ownership
U.S. government, headquarters	15	11	9	2
U.S. government, field	14	11	7	10
Host government, national	5	3	1	3
Host government, subnational	10	9	0	9
Global partner	13	12	11	3
**Total**	**57**	**46**	**28**	**27**

* Some participants were interviewed both on the Governance Framework and for Country Ownership.

### Data Collection

Qualitative interviews were conducted in English in person and by telephone when logistical issues prevented a face-to-face meeting. Interviews were administered by 4 trained qualitative researchers from USAID. While a field guide was used to focus the interviews on our research aims, participants were largely enabled to direct the course of the conversation. All interviews were digitally recorded and notes taken. All interviews were transcribed and loaded onto a secure drive for review. Dedoose qualitative software was used to facilitate the thematic coding process.

### Data Analysis

Each transcript was first reviewed and coded by a primary coder. A second coder reviewed all abstracts and noted disagreements, which were resolved by group consensus. Initial codes were prescribed based on the study aims and expanded upon as themes emerged from the first round of data. Semi-monthly team meetings were held to discuss data concerns, emerging themes, and update the codebook.

### Ethical Approval

The study received Institutional Review Board (IRB) approval from CDC's Center for Global Health (CGHHSR # 2017-222), University of Zambia ERES Converge IRB (FWA00011697), Uganda's Makerere University College of Health Science School of Public Health (IRB00011353), and ICF IRB (FWA00000845) for the global partnership.

## RESULTS

Several recurring themes emerged from our inquiries into the strengths and challenges of the partnership, including: diversity in partner expertise; high-quality monitoring, evaluation, and learning (MEL); strong leadership; lack of clarity in roles and responsibilities; limited representation on the SMGL governance structure; and unbalanced power dynamics (Box).

BOX **Saving Mothers, Giving Life Partnership Strengths and Challenges**

**Strengths**

- **Goal alignment:** Well-aligned, ambitious goal supported high achievement
- **Partner expertise:** Diversity in expertise through the partnership supported a comprehensive program
- **Strength of leadership:** Strong leadership helped support goal alignment and cooperation among partners
- **Strength of monitoring, evaluation, and learning (MEL):** Well-designed, robust MEL strategy supported the use of data for decision making and adaptive management
- **Country ownership:** Achieved through levels of partner coordination and adoption of practices in non-program districts
- **Sustainability:** Achieved through engagement of new partners, sources of funding, and infrastructure

**Challenges**

- **Representation on governance structure:** Some stakeholders were not represented in the governance structure, which affected service delivery
- **Roles and responsibilities – clarity, resources, and organizational structure:** Partner role ambiguity impeded success in some instances
- **Bureaucratic processes:** Issues such as flexibility, continuity of leadership, communication, human resources, and funding mechanisms created implementation challenges
- **Compressed timeline:** Compressed timeline impacted planning, evidence, and funding
- **Perceptions of power:** Unequal power dynamics between partners based on the level of financial contributions affected decision-making ability

### Strengths

#### Goal Alignment

Partner goal alignment was strong. Most respondents identified a 50% reduction in the maternal mortality ratio (MMR) as the primary goal of the partnership. While it was frequently perceived as “very ambitious” or “aspirational,” several respondents suggested the ambitious nature of the goal mobilized commitment and resources that supported program success. One respondent from a subnational host government explained:

It was a very ambitious goal that in the first year [we would have a] 50% reduction in MMR. We looked at people [SMGL partners] and said, “Are you going to make this? This goal is very high.” And they said, “It is good to aim high and then see how things work. At least we got to 30%. And then progressively we'll be able to reduce the maternal mortality ratio by more than 30%.”

The ambitious goal set by the SMGL partnership mobilized commitment and resources that supported program success.

Respondents further noted that partner alignment on the goal facilitated decision making and coordination. One of the global partners commented:

I know that there's the typical sort of bureaucratic challenges, rivalries, funding challenges—all the things that are always inherent in any kind a project. It just seemed like [the partners] really had the mission first and foremost in mind … I think that's one of things that made SMGL function, was that the partners were sort of aligned on the key topline objective.

#### Partner Expertise

Respondents from both USG headquarters and field offices indicated that the diversity of technical expertise and funding was a key strength of SMGL, as illustrated by comments from a field office representative:

I think the partnership was aiming to achieve first of all, having a pool of varied resources. So we have a lot more than if we had one or two people involved, both financial as well as the technical support and understanding. And also just bringing the varied experiences from the different partners, I think, from the very beginning.

Diversity of technical expertise and funding among SMGL partners was one of its key strengths.

The respondents also found that the ability of the private sector to finance efforts directly was useful in filling public funding gaps, along with the ability to fund outside the set funding cycles of government and foundations. The presence of private-sector partners encouraged public-sector partners to consider the private sector when working in-country.

#### Strength of Leadership

Strong USG leadership, particularly from the Secretariat, was highly valued by the respondents. Respondents indicated that the small number of members enabled the Leadership Council to respond quickly to concerns. Partners expressed satisfaction that strong, consistent leadership allowed them to achieve results, such as a uniform maternal and neonatal health reporting system that would support efforts toward mortality reduction in spite of funding gaps and shortfalls. Leadership, in this case, was perceived by many respondents to fill a coordination role rather than a directive role, which supported partnership success. As one global partner explained:

I think that effective leadership made a difference—there was always a sense of team. And that doesn't happen without effort. There was remarkably little ego, which is really hard to do with these separate agencies with their own separate missions coming together for one mission as a team, so a lot of that was just really strong leadership and management, and tone setting, those types of things.

#### Strength of Monitoring, Evaluation, and Learning

SMGL prioritized MEL from the planning phase. The partners jointly developed a results framework and MEL plan that stipulated core indicators to capture through health facility assessments and baseline and endline program evaluations that were regularly tracked. Partners expressed positive sentiments about the creation and use of robust monitoring and evaluation systems at all levels of the partnership, from the community to global level. For example, a respondent from USG headquarters said:

It's kind of a hallmark of SMGL that we don't just produce fluff, we actually provide health outcome data, which is extremely rare in USAID-led projects. I'm very proud of the M&E [monitoring and evaluation] we have done and our ability to work across agency silos capturing outcomes in a really sterling, top-notch way.

Furthermore, a field representative applauded the SMGL's encouragement of country innovations in MEL:

We decided to use our district health systems strengthening approach but with contiguous districts. … So bottom line to me was there was this allowance to allow systems to innovate within the countries of need, which has even happened in Nigeria.

Of the partners that referenced learning, the majority were from respondents in Uganda, who often referenced learning from Zambia MEL findings and using them to improve health outcomes in their own country.

#### Country Ownership

Perceived ownership of SMGL was high at the subnational level in both Uganda and Zambia by the end of the program. Partnership approaches that facilitated country ownership included working within and strengthening the existing health system rather than a parallel structure; engagement of government national and district staff during program design; and alignment of SMGL with existing national health road maps. During a funding gap in the partnership, elements continued through a “relentless, gritty continuation of the approach and the outcomes,” as one USG headquarters respondent explained, attributed to district ownership and leadership. A subnational respondent from Uganda described how active involvement of local players helped to instill a sense of ownership in SMGL activities:

Partners have not done activities in the district without consulting the DHO [District Health Office], the Chief Administrative Officer, and with the Chief Administrative Office, the District Executive Committee. And monthly there have been project coordination meetings and that makes us own whatever we do, that we are implementing in these areas.

The SMGL partnership facilitated country ownership by working within and strengthening the existing health system, engaging government staff during program design, and aligning SMGL with existing national health road maps.

The level of support at the national MOH level was mixed. While the USG and the MOH in Zambia reported national MOH engagement was high, Zambian District Health Office staff and other donors commented on gaps in Zambian MOH engagement at the national level. In Uganda, respondents indicated that additional human and financial resources at the MOH to support and engage in program management would have enhanced country ownership. Still, as one respondent from Uganda explained, successes at the subnational level helped fuel support at the national level:

The district and local leadership were very excited about it. And then when it started to show pretty incredible successes, the government really got behind it, embraced it and wanted to roll it up and package it as one of their everyday work.

#### Sustainability

During the proof-of-concept phase, SMGL front-loaded funding to allow the respective MOH officials time to gradually assume increasing management and financial responsibility for the program. Upfront investments included hiring additional staff seconded to the ministry, purchasing vehicles and equipment, and providing construction and renovation of health facilities and limited commodities. The partnership's decision to build this infrastructure for staffing, transportation, and construction within the existing national MOH system provided sustainable assets that the MOH could build upon moving forward.

Other elements aimed at achieving sustainability and scale-up of the program included incorporating SMGL elements into other USG-supported programs; ensuring MOH staff were involved in project roll-out and thus maintained institutional memory to continue incorporating SMGL elements in government-financed programs; and encouraging new donors to support the SMGL model in their programs. A respondent from Uganda indicated:

A lot of infrastructure improvements have been done … and equipment—those can probably stay longer. Maybe, five years or more. A lot of capacity has been built of the health workers and a number of them have been taken on by the districts of the government of Uganda. They have been put on the government payroll, so I believe with that knowledge that has been passed on to them, that is something that can stay on in the long term.

The Swedish International Development Cooperation Agency (SIDA) provided and continues to provide funds in Uganda and Zambia and to the Global Financing Facility, and the Global Fund to Fight AIDS, Tuberculosis and Malaria. The Belgian Technical Cooperation also continues to provide funds for the SMGL model as of 2018. A respondent from USG headquarters explained:

[In Zambia] the scale up had been quite vigorous and had attracted other financial support. I think that the Swedish aid agency directly provided financial support to districts to implement the SMGL model.

### Challenges

#### Representation in SMGL Governance Structure

Most partners acknowledged that the governance structure made sense on paper (Figure 1), but some felt that the implementation of the committees did not always reflect the diagram, noting underutilization of some partners, unclear roles of specific working groups, or confusion over the value-add of different committees. Some partners mentioned areas of expertise they wished had been represented on the Leadership Council in order to address service delivery gaps on the ground, including midwifery, nursing, water, sanitation, construction, infrastructure, transportation, and supply chain. For example, a Zambian respondent at the subnational level indicated:

They'd say as a district we're having a problem with transportation, but the partner comes with so much resources but cannot meet the one need that will actually impact everything else.

In addition, while partners reported strong district-level engagement in both countries, absence of national MOH representation on the Leadership Council was considered a missed opportunity. A respondent from USG headquarters stated:

They [the MOH] weren't even represented on the Leadership Council in the early days. And I think that was a serious mistake and something that I hope has been corrected and will continue to be corrected. The problem is that you don't have high officials in a host government who are willing to sit through long conference calls or travel to Washington for meetings talking about leadership and governance.

Absence of national MOH representation on the SMGL's Leadership Council was considered a missed opportunity.

#### Roles and Responsibilities: Clarity, Resources, and Organizational Structure

Roles and responsibilities varied widely across partners and included areas of communication, data and analytics, advocacy, program implementation, and procurement. Individual partners and working groups within the partnership frequently reported a perceived lack of clarity in roles and responsibilities. A global partner explained:

The partners were kind of cobbled together pretty quickly, it seemed without a lot of thought of what would they do, how would they contribute in distinctive ways. And that's something that took a long time to resolve, and I'm not sure it even really was resolved.

Smaller global partners noted that it was more difficult for them to make a significant contribution given their limited resources and the ambiguity associated with their role. Two such smaller global partners described:

If I did it all over again, I don't know that I would have entered it [the partnership], only because of our [small] size and scope. I mean, just to think back, and it just seems kind of incredulous to think that we could have contributed more than we did.

I always felt almost bad when we would start celebrating the early reductions that we were talking about, and I don't feel like we really played a super meaningful role because it wasn't set such that we could think about “How can [we] help here? What can we do to play a meaningful role in the goals that have been set here?” … You know they're bigger … bigger budgets, bigger organizations. But we were tiny. So we could sort of stand aside saying, “What do you need us to do? Put us in coach.”

In a few cases, mid-program shifting priorities within a partner organization resulted in reduced compatibility between the organization's mission and partnership needs. Other respondents suggested that large-scale partners possessed an inherent rigidity through their own internal governance structures and organizational objectives, which might have limited their role in the partnership.

#### Bureaucratic Processes

Bureaucratic processes were unique to each partner and sometimes resulted in funding delays, which begot implementation and human resources challenges. Several respondents at the country and global levels noted such challenges:

You can't just hire 'willy-nilly' [haphazardly or spontaneously] just because you have money and you have a program … following these rigid rules of hiring also affected a lot of the timing, in terms of when you can hire. (Zambian partner)

There was a gap of almost 1 year, whereby there was no funding that came into the country. That was again a real challenge and delayed the program for almost a year. (Ugandan subnational partner)

I think in any partnership, a funding cycle has different sorts of decision makers and timelines, and I would not say we were fast. There were several delays in our funding, but it was often [because], you know, we didn't have congressional approval or things like that. So it's hard to control but it's the reality of how funding gets allocated. (Global partner)

Leadership turnover during political transitions impeded strategy and vision alignment across partners. One partner in Zambia, for example, discussed the difficulties of hiring short-term employees to fill human resource gaps due to discordance between local labor laws and unpredictability of USG funding. While many partners acknowledged that bureaucracy is inherent in government partnerships, some suggested that the private sector was not fully leveraged to counterbalance this challenge and that the partnership should have sought funding with more flexibility. One global nonprofit partner explained the type of flexibility it had in contrast with larger organizations:

It's a lot easier for a tiny little startup non-profit to say, “We're deciding where we're going to spend our money based on what we decide we want to do, so yeah, tell us where to put it.” We could do that. I think for these bigger organizations it's just unrealistic to think that they could [do that].

#### Compressed Timeline

The desire to launch activities quickly and to show significant impacts in a brief period of time seemed to undercut planning and relationship development. Some members found themselves playing “catch-up” after implementation began, finding it necessary to insert themselves into a moving process, rather than taking position in a prearranged operational structure at the outset of program activities. One partner noted the challenge posed by the initial short timeline to gathering sufficient evidence, as countries were not guaranteed additional financial support unless they had achieved significant reductions in MMR during that year. Another partner explained:

I think some of the ground work that would ordinarily happen when trying to put together a partnership of this size, it just didn't because speed seemed really important. There's this real desire to get something off the ground quickly, and so there wasn't the planning and the groundwork that you would usually see with something like this until it was catching up and learning more information, figuring out how to plug in. So it wasn't the ideal dynamic.

#### Perceptions of Power

Financial capacity affected power dynamics in several ways. In at least one case, a partner on the Leadership Council was financially supported by another council partner. Partners of both public and private sectors observed that those with larger financial contributions had more decision-making power. Since USG invested more money than other organizations, this shifted more power to USG partners. One of these USG global partners explained:

Huge decisions like how many years to keep SMGL going [were] largely driven by funding. … I think the USG held a huge role in decision making because we had the big purse.

SMGL partners with larger financial contributions had more decision-making power.

Other partners described this imbalance with the terms “big P” and “small p” partners to illustrate the functional differences between certain partners:

[A]nd by big P [Partner] and small p [partner], it had to do with who had the biggest investments and therefore gets the biggest seat at the table. So that was a little bit concerning for us, because those big P partners seemed to have had more of the say in the partnership.

Generally, USG partners felt that the funding level of an organization reflected its level of commitment and hence determined its ownership. Some USG partners even questioned the value of including non-USG partners, arguing that the administrative burden outweighed their added value.

## DISCUSSION

Studies have clearly found that SMGL significantly reduced maternal mortality, and to a lesser extent perinatal mortality, in Uganda and Zambia.^11^^,^^12^ The current qualitative study reported on in this article aimed to shed light on remaining questions about the importance of using a partnership approach, whether the outcomes justified the means, and whether these efforts could be owned and sustained by local stakeholders, given the large influx of donor funding to achieve these results. We summarize our findings on overall partnership success factors, the governance structure, and country ownership and sustainability and place the findings in context of the partnership literature.

### Overall Partnership Success Factors

Evaluations of large-scale global health PPPs, including Gavi, The Global Fund to Fight AIDS, Tuberculosis and Malaria, Roll Back Malaria, the International AIDS Vaccine Initiative, Stop TB Partnership, the International Partnership for Microbicides, Medicines for Malaria Venture, and the Global Alliance for Elimination of Leprosy, have reported the lack of a strategy and unclear roles and responsibilities as major challenges to partnership success.^2^^,^^3^^,^^13^^–^^15^ Another study found that launching prematurely and without a strategy were key perils of the 15 multi-stakeholder partnerships they reviewed.^14^ As previously mentioned, the SMGL Phase 1 evaluation, conducted 1 year after SMGL implementation, found the lack of an agreed-upon operational and financing plan hindered effectiveness of the partnership and complicated future planning.

Given that the goal established by then-Secretary Clinton was to reduce MMR by 50% within a year, there was pressure on all partners to demonstrate results in a short time period. This meant that the design and planning process was truncated to quickly start implementation and demonstrate results. Frustration was generated when SMGL funding was guaranteed for only 1 year with subsequent support based on achievement of reductions in maternal mortality within a short-time frame. Any future substantial systems approach focused on maternal and neonatal mortality reduction should commit to a minimum of 5 years of support from the beginning. Our findings suggest both relationship building and evidence gathering takes time and partnerships would be well-served to structure their funding and planning strategies with these critical components in mind.

Maternal and neonatal mortality reduction efforts should commit to a minimum of 5 years of support from the outset.

Despite the pressure to develop the partnership quickly and achieve ambitious results, our data suggest that the SMGL partnership was able to overcome many of these initial challenges (Table 3). For example, the partners were able to develop a mutually agreed-upon operational and financing plan, which helped clarify roles and responsibilities during Phase 2. This is owed, in part, to the governance structure and the responsiveness of the Leadership Council to integrate monitoring and evaluation activities. In addition, partners indicated willingness to be flexible in their roles to address issues as they arose. USG interagency collaboration and clarity of roles can often be challenging. SMGL seems to have found the right balance for effective coordination that could be used as a model for other interagency initiatives. Former U.S. Ambassador to Zambia Mark Storella reported that SMGL in Zambia was “one of the best team-building experiences I had as a diplomat, we built cross-agency teams that fostered on the ground collaboration.”^18^

**TABLE 3.** Saving Mothers, Giving Life Partnership Strengths and Weaknesses Compared With Overall Partnership Success Factors

Success Factors	Summary of Partnership Literature^2^^,^^3^^,^^13^^,^^14^^,^^16^^,^^17^	SMGL Findings	
		Strengths	Weaknesses
Shared vision/operational approach	At the vision level, there are often high levels of agreement, but it is more challenging to align operational approaches and resources.	The partners had a shared vision in terms of reducing maternal and newborn deaths. Initially, operational approach was not clear, but the partners successfully negotiated a mutually agreed-upon operational approach and budget.	Country governments had limited input in developing the initial goal, but goal expectations were later modified. Partners assumed it was easy to integrate PEPFAR and MCH platforms.
Trust	Gaining trust takes time and initially relies on personal connections. Staff changes can significantly destabilize a partnership.	While there were many changes in the partnership, organizations continued their commitment to the partnership, even if at a lower funding level.	The rapid startup limited time at the outset to develop trust and define roles and responsibilities.
Clearly defined roles and responsibilities	Often, lack of clarity in roles and responsibilities can delay activities, create duplication, waste resources, and lead to miscommunication/mistrust among the partners.	As the operational plan was clarified, the roles and responsibilities became clearer.	Initially, there was confusion over roles and responsibilities, which was particularly challenging for some of the smaller partners.
Resources	The partnership can mobilize additional resources, but often fails to be suffciently resourced to meet ambitious goals. There are high transaction costs. Due to inadequate use of country systems and poor harmonization, resources can be duplicated/wasted. Pledges are not always been realized.	The partnership facilitated the use of PEPFAR funds for maternal health activities. Presence of a private-sector partner provided more engagement with private service providers. Additional partners were leveraged to fill gaps and expand the approach.	The initiative was not fully funded, partners had to revise their pledges and recommit themselves to the partnership. The partnership was limited in its capacity to provide infrastructure support.

Abbreviations: MCH, maternal and child health; PEPFAR, U.S. President's Emergency Plan for AIDS Relief.

### Partnership Governance

Global health PPPs often experience tensions between the perceived urgent need for results and adequate commitment to and investment in capacity of governance mechanisms to effectively manage these complex structures. Roehrich found that issues of incentivization, stakeholder trust, optimal balance of skills and capabilities, and information and power asymmetry can impact stakeholder alignment in PPP arrangements and, ultimately, program success.^17^ Partners often do not understand the pressures and incentives faced by different partners that can interfere with overall functioning and effectiveness of a partnership.^3^ The human resource capacity within a partnership's secretariat has also been found to be critical in determining its success.^3^^,^^14^

According to Buse, a good governance structure including the right constellation of partners and its modus operandi are essential to the success of a partnership.^3^ However, there are often tensions between ensuring adequate inclusivity and establishing a manageable quorum to effectively operate and make timely decisions. A system of accountability among partners is increasingly important and formalization of global health PPP governance structures is a must, but formalization needs to be balanced with the flexibility to respond to challenges and opportunities, particularly at the country level.^13^

Other global health PPP evaluations have identified key challenges as^2^^,^^3^^,^^13^^–^^15^:

- Poor governance practices, including conflicts of interest
- Limited voice in decision making, particularly from host-country officials
- Limited focus on health systems
- Unclear performance metrics
- Poor understanding of the costs and benefits
- Insufficiently resourced arrangements to implement activities and pay for coordination costs
- Poor harmonization with governments and other development partners

Table 4 contrasts these challenges with our results.

**TABLE 4.** Saving Mothers, Giving Life Governance Strengths and Weaknesses Compared With Established Success Factors

Success Factors	Summary of Partnership Literature^2^^,^^3^^,^^13^^,^^14^	SMGL Findings	
		Strengths	Weaknesses
Governance structure	Low participation from countries and NGOs on governing bodies but boards are becoming more representative. Partnerships require dedicated staff to support them.	The partnership developed a defined governance structure with voting and clearly identified organizational points of contact. Composition size was seen as a positive.	MOHs were not included on the Leadership Council during Phase 1. They were invited to join during Phase 2, but country factors inhibited their participation.
Secretariat	The Secretariat plays a vital role in the effectiveness of the partnership; the costs of coordination and communication are often not well understood or resourced.	The Secretariat provided stability to the partnership and was praised for its leadership.	
Governance process: M&E	Agreement on common metrics, data collection approaches, and partner roles are essential. It is important to have indicators that reflect the outcomes as well as the partnership processes.	Rigorous M&E enabled the partnership to demonstrate success and make program adjustments.	The Phase 1 evaluation touched on the partnership, but the partnership did not have any metrics that measured the partnership processes.
Governance process: decision making	Dominant decision makers are usually related to the size of funding.	Regular (technical, results, and financial) updates were provided via the Operations Committee and Leadership Council.	The partnership was largely seen as Washington-driven and USG-funded. There were some conflicts of interest and power dynamics between larger and smaller partners.

Abbreviations: M&E, monitoring and evaluation; MOH, Ministry of Health; USG, United States Government.

Respondents indicated that SMGL had very robust health outcome metrics but lacked measures to assess partnership processes; was well-aligned with national government policies, including focusing on the public and private health system; and generally had an effective governance structure and processes. Like other global health PPPs, SMGL struggled to get full participation of national governments on the formal governance structure; fully address some of the power dynamics between the larger and smaller organizations; and fully realize financial commitments. While respondents generally felt the composition of the governance structure was appropriate, key skills, such as infrastructure, were missing. With that said, the partners were creative and flexible in responding to issues as they arose, refocusing efforts on 3 countries and leveraging an additional US$100 million outside of the partnership to expand the approach.

### Country Ownership

As previously mentioned, while not explicitly included as a goal, country ownership and sustainability were key tenets of SMGL's approach. The GHI framework articulated 4 aspects of country ownership that needed to be addressed: (1) political stewardship; (2) institutional and community ownership; (3) capabilities; and (4) mutual accountability and financing.^19^ Table 5 compares key elements of country ownership and sustainability with our results.

**TABLE 5.** Saving Mothers, Giving Life Country Ownership and Sustainability Strengths and Weaknesses Compared With Established Success Factors

Success Factors	Summary of Partnership Literature	SMGL Findings	
		Strengths	Weaknesses
Country ownership^20^^–^^24^	Country ownership of partnership activities can strengthen national health policy processes, raise profile of specific health issues, and establish international norms and standards. Partnerships often fail to address broader health systems issues. Limited harmonization leads to considerable duplication, emergence of parallel systems, and little alignment between recipient country and partnership priorities. Parallel budget systems raise concerns of government ownership and sustainability.	SMGL activities were built on national policies/road maps and international best practices. The partnership reinvigorated commitments to reducing maternal/newborn deaths. The partnership focused on enhancing district health systems, both public and private, to achieve results. SMGL built health worker and community capacity to increase demand for and provision of quality maternal and newborn health services.	Rapid startup limited initial government ownership. Some misalignment between partners and country priorities existed.
Sustainability^25^^–^^29^	Transition planning is key but not sufficient. Ensuring financial sustainability is the most challenging aspect of partnerships; it is important to understand the cost of the entire system to be sustained, rather than just commodities. More studies and indicators to monitor successful transitions from donor-funded programs to country, public, civic, and/or private stakeholders are needed.	SMGL was designed to front-load funding so the MOH and other stakeholders could sustain the efforts. Communities and some districts were able to mobilize their own resources. The partnership between the MOHs and SMGL leveraged US$100 million from donors to continue key aspects in the short run.	Partners used its results to advocate with key government stakeholders to sustain SMGL. While there was a high level of government ownership for SMGL, this did not result in national-level budget increases.

Abbreviations: MOH, Ministry of Health; SMGL, Saving Mothers, Giving Life.

SMGL was able to apply some lessons from global health PPPs started in the early 2000s. One area was to begin the discussions about country ownership and sustainability at the onset of the partnership. SMGL was designed to front-load funding to demonstrate a successful model in the first year that the MOH and other donors and constituencies (e.g., the private sector) could adapt and expand. This was thought to provide time for the MOH to gradually assume more responsibility and financing for the program, as SMGL's resources would decline. As with other global health PPPs, SMGL has been quite successful in garnering government ownership over time, particularly at the district level, as well as community and provider buy-in. This has been combined with substantial increases in district-level capacities, especially in data analysis and use and in quality of care, which contribute to improvements in the health system overall.

Discussions about country ownership and sustainability began at the onset of the SMGL partnership.

Despite early indications that the SMGL approach was supported but not “truly owned” by governments,^10^ our results provide clear examples that both governments, particularly at the district level, have adopted key elements of the SMGL approach and have encouraged other donors to use this model. Both Uganda and Zambia have expanded elements of the SMGL approach, with MOH funds as well as with other donor support, beyond the initial districts.

### Sustainability

Sustainability aims to systematize and institutionalize the 4 country ownership domains, described above, so that they become usual practice within the host country health system. While there is a large body of literature on sustainability, there is limited data on metrics to track progress of the transition of large-scale donor health programs to local counterparts.^26^ In addition, there are few examples of PPPs that have been sustained at a country level. In an effort to promoting self-reliance, USAID has successfully transitioned its family planning and reproductive health programs in several countries. Key transition domains for such graduation include leadership, financing, programming, and service delivery. Activities that support the transition include sustaining a supportive policy environment, creating financial sustainability, developing local stakeholder capacity, communicating to all stakeholders, and aligning programs.^26^

Experience has found that poorly executed transitions of large-scale donor programs can reverse health gains. Two relevant studies for comparison with SMGL are the transition of the Gates' funded Avahan HIV program in India,^27^ not a PPP, and the Gavi graduation model.^28^ The review of the Avahan program found that while transition readiness among local stakeholders was important, it did not necessarily lead to institutionalization of key program elements after the 1-year transition period. In addition, institutionalization was not predictive of sustained program delivery.^27^ For Gavi, political commitment was a crucial factor, particularly to increase and sustain immunization budgets. The larger the budget increase required, the more difficult it was for the country to secure financing. It was also important to ensure that the investment envelope included the total cost of the system rather than selected elements. Lastly, the expectation that a country will have to pay a greater share of the program costs over time allows for transition planning to start early in the partnership. Transition time is needed to (1) secure buy-in from multiple stakeholders; (2) ensure capability of structures and processes; and (3) finalize the funding mechanism(s) to mainstream the initiative.^28^

Financial sustainability is the most challenging for all programs, not just global health PPPs. SMGL was not designed to specifically increase national maternal health budgets, but it was anticipated that the programmatic results would drive change and could be used to advocate for increases in domestic resources. There are examples where some districts, facilities, and/or communities have been able to raise some local resources and continue key practices (e.g., better data analysis) without additional resources. Both Ugandan and Zambian MOHs were active, in collaboration with SMGL, in encouraging other donors to support many of the SMGL-supported activities so they would be sustained for years after the partnership. The future of long-term financing is affected by multiple factors and thus unclear at this time. Unfortunately, the Ugandan MOH's budget has been reduced in the past year. (For additional analysis and discussion on SMGL sustainability, see the companion article by Healey and colleagues^29^ in this supplement.)

### Limitations

There were several limitations to our study. First, while we had an 80% response rate, scheduling conflicts reduced the participation from senior Zambian MOH officials who could have provided valuable insights. Second, the team chose to exclude implementing partners from this study, because they did not participate in the SMGL governance structure and were not specifically selected for the partnership. Furthermore, given that SMGL used existing mechanisms, the implementing partners involved in the proof-of-concept phase transitioned to other implementing partners as the USG agreements were procured. However, this decision was not without sacrifice, as the partnership structure can have implications for implementing organizations. Lastly, the study team was comprised by USAID staff. The team took every measure to conduct the study with integrity and to maintain fidelity to the research and analytic processes prescribed and minimize biases. To address the latter, qualitative interviews and data analysis were conducted by USAID staff who had no prior experience or affiliation with the SMGL project. Respondents were informed before each interview that accuracy was our objective, that the interviewer was not otherwise affiliated with the SMGL project, and that the respondents' identity would remain anonymous. Respondents did not seem to hold back critical commentary related to USAID or other USG partners.

## CONCLUSIONS

This qualitative study found that representatives of the SMGL partnership believed that the partnership approach, in part due to its diversity, supported the achievement of SMGL's results. The partnership faced many of the same challenges experienced by other global health PPPs, but local counterparts and SMGL partners were able to successfully address many of these issues. Despite agency bureaucracy, SMGL was praised as a successful model for interagency coordination. Examples of country ownership and short-term financial sustainability have been put in place for many elements of the SMGL approach. Long-term financing is still a challenge for SMGL as well as other global health PPPs.

Given the importance of country ownership and response to local context, future global health PPPs should have greater focus at the country level, ensuring diverse representation of local stakeholders in partnership governance structures. In addition, global health PPPs should include regular self-assessments or reflective learning processes, with clear metrics, of the governance structure and its processes to reduce transaction costs and increase efficiencies, ultimately enhancing the effectiveness of the partnership to deliver even greater results.

Global health public-private partnerships should have greater focus at the country level and ensure diverse representation of local stakeholders in governance structures.

The SMGL partnership was an ambitious attempt to dramatically reduce maternal and newborn deaths in just a few years through the strategic, cooperative efforts of government and private organizations with a shared goal. Its legacy will provide that strong leadership, a broad alliance of stakeholders, integrated monitoring and evaluation, and agile implementation can achieve dramatic results in global health.

## Notes

**Peer Reviewed**

**Funding:** Saving Mothers, Giving Life implementation was primarily funded by the Office of the Global AIDS Coordinator, the U.S. Agency for International Development (USAID), Washington, D.C., the Centers for Disease Control and Prevention (CDC), Atlanta, Georgia (Cooperative agreements GPS003057 and GPS002918), Merck for Mothers, and Every Mother Counts. The funding agencies had no influence or control over the content of this article.
